# Inclusion of eye medication in national health care systems

**Published:** 2023-05-22

**Authors:** Junu Shrestha, May Ho, Jude Stern

**Affiliations:** Policy & Advocacy Manager: International Agency for the Prevention of Blindness, London, UK.; Optometry & Primary Care Adviser: The Fred Hollows Foundation, Melbourne, Australia.; Head of Knowledge Management: IAPB, Sydney, Australia.

**Advocacy for eye medicines is easier with these helpful resources and guidance.**

**Figure:**
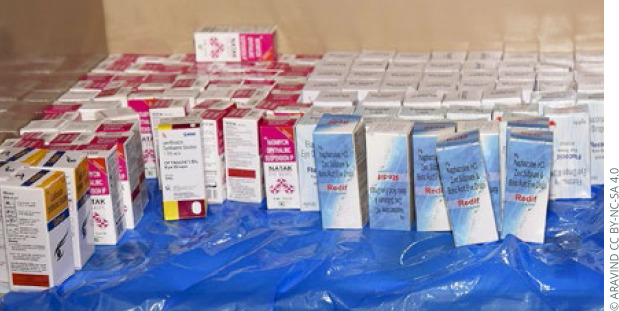
With so many eye medicines available, the WHO model list provides helpful guidance.

The World Health Organization (WHO) maintains a model list of essential medicines. The essential medicines include those that satisfy the priority health care needs of a population. The medicines are the most effective, safe, evidence-based available and are comparatively cost-effective. They are intended to be available in health systems at all times. WHO recommends that countries make these medicines available in the appropriate form and dosage, and ensure that they are available, accessible, and affordable to everyone in need. Universal access can only become possible only when medicines are included in a country's essential medicines list and funded by the national health financing system.

The WHO model list of essential medicines includes ophthalmic medicines in section: 14.1 diagnostic agents: ophthalmic medicines; and section 2: ophthalmological preparations. This information needs to be communicated to the policy makers and referred when advocating for universal eye health. The latest list is available here: bit.ly/WHO-em

The WHO's Package of Eye Care Interventions (PECI), launched at the World Health Assembly in 2022, is a set of evidence-based eye care interventions and the resources needed for their implementation. PECI – which includes the list of ophthalmic medicines in the WHO essential medicines list – is designed to support policy makers and technical decision makers to integrate eye care into the health care services system of a country. This tool is an important resource when advocating for the inclusion of essential eye medicines in a national essential medicines list and in health financing benefit packages.

When advocating for eye medicines, also refer to the United Nations’ Sustainable Development Goals, target 3.8. This target focuses on achieving universal health coverage, including financial risk protection, access to high quality essential health care services and access to safe, effective, high quality affordable essential medicines and vaccines for all. Without provision for equitable access to essential medicines for eye conditions, achieving universal eye health coverage is not possible.

Access to essential ophthalmic medicines also aligns with the principle of integrated people-centred eye care (IPEC). The IPEC was adopted by the 73rd World Health Assembly resolution in 2020. To know more about advocating for IPEC, check out IAPB's IPEC Advocacy to Action Toolkit. The toolkit includes PowerPoint slides, letter and IPEC policy brief templates that can be adapted and used to approach stakeholders for policy dialogues.

The WHO has also produced guidelines on using the WHO Model List of Essential Medicines to update the national essential medicines list. See https://bit.ly/useWHOem

